# Dairy calcium intake and lifestyle risk factors for bone loss in hiv-infected and uninfected mediterranean subjects

**DOI:** 10.1186/1471-2334-12-192

**Published:** 2012-08-15

**Authors:** Valentina Li Vecchi, Maurizio Soresi, Lydia Giannitrapani, Giovanni Mazzola, Sara La Sala, Fabio Tramuto, Giuseppe Caruso, Claudia Colomba, Pasquale Mansueto, Simona Madonia, Giuseppe Montalto, Paola Di Carlo

**Affiliations:** 1 Dipartimento di Medicina Interna, Università di Palermo, Via del Vespro 141, Palermo, I-90127, Italy; 2 Dipartimento di Medicina Clinica e delle Patologie Emergenti, Azienda Ospedaliera Universitaria Policlinico “Paolo Giaccone” di Palermo, Via del Vespro 133, Palermo, I-90127, Italy; 3 Dipartimento di Scienze per la Promozione della Salute, Università di Palermo, Via del Vespro 133, Palermo, I-90127, Italy; 4 Dipartimento di Biotecnologie Mediche e Medicina Legale, Università di Palermo, Via del Vespro 133, Palermo, I-90127, Italy

**Keywords:** HIV, Osteopenia, Osteoporosis, Dairy intake, Bone mineral density

## Abstract

**Background:**

Despite the reported high prevalence of osteoporosis in the human immunodeficiency virus (HIV)-population, there have been no previous studies examining dairy calcium intake and bone mineral density (BMD) in HIV-subjects.

We assessed the prevalence of low BMD in HIV-infected and uninfected subjects and analyzed the effects of calcium intake, lifestyle and HIV-related risk factors on BMD.

**Methods:**

One hundred and twelve HIV-infected subjects were consecutively enrolled. Seventy- six HIV-uninfected subjects matched for age and sex were enrolled as the control group. The HIV-subjects were interviewed about lifestyle habits and completed a weekly food-frequency questionnaire to estimate calcium intake. HIV-RNA, CD4+ T-cell count and data on antiretroviral therapy were also recorded. Both biochemical bone turnover markers and BMD, assessed by dual-energy radiographic absorptiometry (DXA) were recorded in the HIV-cases and controls. We also calculated the 10-year fracture risks using the WHO FRAX equation.

**Results:**

Osteoporosis prevalence was significantly higher in the HIV-cases than controls (*p* < 0.05). BMI values were positively correlated with BMD (*p* < 0.05). Vitamin D levels were lower in the HIV-subjects (*p* < 0.02). No correlation was found with daily calcium intake.

BMI values were significantly correlated with dairy intake quartiles (*p* < 0.003). In HIV-subjects, the mean of FRAX score was 1.2 % for hip and 4.7 % for major osteoporotic fractures. On multivariate analysis of the lumbar spine DXA T-score, age (*p* < 0.005) and HIV/hepatitis C virus co-infection (*p* < 0.0001) were negatively correlated with BMD, while yogurt intake was a protective predictor of BMD (*p* < 0.05). In the femur DXA T-score, age (*p* < 0.01), nadir CD4 + T-cell count < 200 cells/μL (*p* < 0.05) and drug addiction ( *p* < 0.0001) were negatively correlated with BMD.

**Conclusions:**

Among the foods rich in calcium, yogurt was a protective predictor of BMD in HIV-subjects. HIV/HCV co-infection, nadir CD4 + T-cell count < 200 cells/μL and drug addiction were independent predictors of severe BMD. Promoting behavioral changes in food intake and lifestyle, aimed at the primary prevention of bone disease in the chronically-infected subjects seems to be essential for implementing medical intervention in these cases.

## Background

Osteoporosis and its resulting fractures are becoming major public health issues due to population ageing and the world-wide increase in long-term chronic conditions [1-3].

However, fractures in different populations considered at low risk of osteoporosis and with low-normal bone mineral density (BMD) have also been reported [4,5].

Low BMD has emerged as a significant problem in chronically-infected patients, such as the HIV-infected population [1-3]. The mechanisms by which risk factors accelerate bone loss in human immunodeficiency virus (HIV)-infected subjects are unclear because of their complex and multifactorial nature [6]. The chronic inflammatory state induced by the virus itself may promote bone loss by increasing levels of pro-inflammatory cytokines such as tumor necrosis factor-α (TNF-α), IL-6, IL-1 and NF-kappaB-ligand (RANKL), a member of the TNF superfamily of ligands and receptors [1,6]. Also weight loss, hypoparathyroidism and vitamin D deficiency resulting from HIV infection, as well as many antiretroviral drugs, may promote bone loss [7].

In addition to the traditional risk factors (i.e. older age, female sex, menopause, corticosteroid therapy, low BMI) attention has recently been centered on the importance of following a diet rich in calcium, other minerals and vitamin D associated with physical activity, which is recommended to maintain not only a healthy bone structure but also an acceptable BMD [8].

Since general risk factors and HIV-related factors affect both bone turnover and BMD, the aim of our study was to assess the prevalence of low BMD in HIV-infected subjects and controls and to analyze the effects of calcium intake and other environmental risk factors in order to increase current interest in the management of bone disease in the HIV-population through appropriate dietary and lifestyle interventions. We also estimated the 10-year fracture risk.

## Methods

### Study population

The study participants were Caucasian HIV-infected patients, consecutively enrolled between January 2011 and January 2012, who were being followed-up prospectively at the AIDS Center of the University of Palermo.

BMD was measured in all subjects by dual X-ray absorptiometry (DXA) [9]. Exclusion criteria included kidney disease, gastrointestinal disorders and steroid and sex steroid therapy.

Data on age, gender, drug addiction, antiretroviral therapy (ART), protease inhibitors (PI) and non-nucleoside reverse-transcriptase inhibitor (NNRTI) exposure were all recorded in a database designed for this study. Specific tenofovir exposure > 1 year was also recorded. Exposures to ART and PI were graded 0 (no exposure) to 3 (1: < five years; 2: from five to nine years; 3: ≥ ten years).

Body mass index (BMI) was calculated as weight (in kg) divided by height squared (m^2^). Subjects were considered underweight when BMI was ≤ 18.5 kg/m^2^, normal weight when BMI was between 18.6-24.9 kg/m^2^, overweight when BMI was between 25–29.9 kg/m^2^ and obese when BMI was ≥ 30 kg/m^2^.

Diabetes mellitus was defined in accordance with the Expert Committee on the Diagnosis and Classification of Diabetes Mellitus criteria [10].

Seventy-six HIV-uninfected subjects referred by family doctors to our Center for laboratory and BMD assessment were enrolled as the control group. Age and sex of these subjects were comparable with the patients and the same exclusion criteria were applied.

### Interview data

At the start of the study, the HIV-infected subjects filled in a health and lifestyle questionnaire considering medical and drug abuse history, lifestyle habits (including smoking status, alcohol consumption, Italian espresso coffee drinking, physical activity) and bone fractures. Smoking status was categorized as non-smokers, former smokers and current smokers. Alcohol intake > 20 g per day either at the time of the study or in the past as well as Italian espresso coffee intake > 4 cups per day were also recorded. Physical activity was defined as either non-occupational or occupational when physical exercise was performed during free time or at work, respectively. Non-occupational physical activity was considered active when > 2 sessions of 20 minutes per week were performed. Occupational physical activity was defined as heavy when manual work was carried out.

In order to assess calcium intake, the consumption of foods representing the major sources of daily calcium intake in the Italian diet, such as typical Italian aged cheese (i.e. Parmesan), ricotta cheese and yogurt was recorded in a weekly food-frequency questionnaire, in accordance with the tables of nutrient values issued by the Italian National Institute of Nutrition [11-13]. This was administered through an individual face to-face interview [11]. A fixed range of food containers, i.e. a glass for milk and a cup for yogurt, was used to standardize portion sizes, each containing ≈ 300 mg of calcium. A similar amount of calcium was contained in the reference servings of aged and ricotta cheese (≈ 25 and 100 g, respectively). Color photographs were shown to the subjects to demonstrate the standard sizes of the cheese servings. In addition, the number of servings eaten weekly was recorded and calcium intake was categorized according to quartiles of weekly servings (1: ≤8, 2: 9–10, 3: 11–14, and 4: ≥15), based on the distribution of the HIV-population. The level of education was also determined for all subjects. Three options were used: primary school (which in Italy represents a five-year compulsory education period), lower secondary school (three additional compulsory years) and higher levels of education, including upper secondary school, professional schools or university.

### Laboratory methods

In all the HIV-patients CD4+ T-cell count (most recent value and nadir) and plasma HIV-RNA levels were assessed as previously reported [14]. Serum bone alkaline phosphatase, 25-hydroxyvitamin D, phosphorus and calcium levels were obtained in both HIV -infected cases and un-infected controls.

Vitamin D assessment was performed in the sunnier months from April through October, since it is known that vitamin D formation is not possible or inadequate [15] in Italy during the less sunny months, due to its latitude.

### Bone mineral density assessment

For both HIV-infected cases and uninfected controls, BMD was assessed at baseline by DXA, using a QDR Discovery Hologic DXA in the femoral neck and DXA in the lumbar spine by total body DXA [16]. For each scan, BMD and T-scores were recorded. T-scores compare BMD with the mean of a healthy young (age 20–30 years) reference population, matched for sex and race, and were expressed as the number of standard deviations above or below the reference mean. Osteopenia was defined when at least one of the two DXA T-scores was less than −1. Osteoporosis was diagnosed when either femoral neck or lumbar spine DXA T-scores were less than −2.5, as recommended by the World Health Organization (WHO) and the National Osteoporosis Foundation [17-19].

We calculated the 10-year fracture risks according to the standardized WHO FRAX equation, computed with BMD (T-score) at the femoral neck [20].

### Statistical analysis

Data were expressed as mean ± standard deviation when distribution was Gaussian. Differences were calculated using Student’s t-test. Otherwise, data were expressed as median and range and analyzed with the Mann–Whitney U test. Fisher’s exact and χ^2^ tests, Pearson’s correlation and Spearman's rank correlation were used where appropriate. Multiple logistic regression analysis was performed to estimate the independence of the association between lumbar spine and femoral neck T-scores as well as variables significant at univariate analysis in the HIV-infected subjects. Variables contributing significantly to fit the logistic equation were then selected by a step-wise procedure. *P* < 0.05 was considered significant.

All analyses were performed using the SPSS software package (version 16.0; Chicago, IL, USA).

The study protocol conformed to the ethical guidelines of the 1975 Declaration of Helsinki.

The study was approved by the local Ethics Committee and informed consent was obtained from all subjects.

## Results

### Characteristics of the HIV-infected population

The main characteristics of the 112 HIV-infected and control subjects are shown in Table 1.

**Table 1:** Main characteristics of the 112 HIV-infected and 76 HIV-uninfected subjects

	**HIV+**	**HIV-**
**Demographic and lifestyle characteristics**		
Age years ± SD	47 ± 9.7	49 ± 11.3
Sex (M/F)	63/49	42/34
BMI (kg/m^2^):		
**<** 25	77 (68.7)	47 (62)
≥ 25-29	28 (25)	23 (30)
≥ 30	7 (6.2)	6 (8)
Smoking status:	37 (33)	
*Non- smokers*		44 (58)
*Smokers*	58 (53)	21 (28)
*Former smokers*	16 (14)	11 (14)
Free time physical activity	57 (51)	45 (60)
Heavy workers	16 (14)	10 (13)
Italian espresso caffeine intake^1^	30 (27)	23 (29)
Alcohol	6 (5)	3 (4)
Diabetes	20 (18)	11 (14)
Education:		
*Primary school*	34 (30.3)	20 (26)
*Secondary school*	34 (30.3)	26 (34)
*Higher level*	44 (39.3)	30 (40)
Postmenopause	18 (36.7)	26 (34)
Fracture	4 (3.5)	2 (3)
**Calcium intake**		
Daily calcium intake (mg/day) (range)	454 (96-1359)	
Dairy intake (times/week) (range)	10 (2-32)	
Daily milk intake (ml/day) (range)	200 (0-800)	
Yogurt intake	36 (32)	
Yogurt intake (ml/week)	0 (0-1750)	
Parmesan intake	100 (89)	
**Characteristics of HIV infection**		
Time since HIV diagnosis (years) (range)	15 (1-27)	
Drug addiction	28 (25)	
CD4+ T-cell count (cells/μL) (range)	551 (5-1082)	
nadir CD4+ T-cell count <200 (cells/μL)	75 (67)	
Undetectable HIV-RNA	81 (72.3)	
ART	104 (93)	
Duration of ART exposure:		
< 5 years	18 (17.3)	
5-9	17 (16.3)	
≥ 10	69 (66.3)	
HCV infection	41 (36.6)	

^1^ Italian espresso caffeine intake = > 4 cups per day.

The average age of the recruited patients was 47 ± 9.7 years. Sixty-three individuals were male (56%). Among the 112 subjects, 28 (25%) were overweight, 6 (5%) were underweight (BMI < 18.5) and 7 (6%) were obese. As regards smoking status, drug addiction and Italian espresso coffee drinking we found that half of the study subjects regularly smoked cigarettes, one third drank coffee > 4 times a day and one third used cocaine and/or heroin.

About half of this population did physical activity in their free time and had a higher level of education.

Concerning eating habits, the median daily calcium intake was 454 mg/die, range (96–1359). More than half of the participants interviewed reported that they drank a glass of milk in the morning, whereas yogurt intake was less frequent. Parmesan cheese intake, according to our definition, was reported in about 90% of subjects.

Most subjects were on ART (93%), seventy-five (67%) had nadir CD4+ T-cell count < 200 (cells/μL) and 41 (36.6%) were co-infected with hepatitis C virus (HCV) (Table 1).

### Prevalence of osteopenia and osteoporosis in HIV-infected and uninfected subjects

Osteopenia was present in 38 (34%) HIV-infected vs 19 (25%) HIV-uninfected subjects (*p* = ns). In detail, at the lumbar site osteopenia was present in 37 (33%) HIV-infected vs 17 (22%) HIV-uninfected subjects (*p* < 0.02); at the femoral site osteopenia was present in 42 (37.5%) HIV-infected vs 18 (24%) HIV-uninfected subjects (*p* = ns). In the setting of HIV-infection, osteopenia was present in 18 subjects at both the lumbar and femoral sites.

Osteoporosis was present in 34 (30.3%) HIV-infected vs 8 (10%) HIV-uninfected subjects (*p* < 0.05). At the lumbar site osteoporosis was present in 34 (30%) HIV-infected vs 8 (10%) HIV-uninfected subjects (*p* < 0.05); at the femoral site osteoporosis was present in 9 (8%) HIV-infected vs 4 (5%) HIV-uninfected subjects (*p* = ns).

In the setting of HIV-infection osteoporosis was present in 9 subjects at both the lumbar and femoral sites.

### Comparison between lumbar spine and femoral neck DXA T-scores and biochemical parameters in HIV-infected and uninfected subjects

Lumbar spine DXA T-score were significantly lower in HIV-infected (−1.4 range −5.2 to 1.6) than in uninfected subjects (−1 range −4.4 to 2.7) (*p* < 0.02). No significant difference was found in femoral neck DXA T-score in patients with or without HIV-infection (data not shown).

Serum bone alkaline phosphatase was significantly higher in HIV-infected (54.7 range 19–170 IU/L) than uninfected subjects (28.2 range 5–84 IU/L) (*p* < 0.0001), while phosphorus levels were lower in HIV-infected (3.1 range 1.4-4.4 mg/dL) than in uninfected subjects (3.5 range 2.1-6.8 mg/dL) (*p* < 0.0001). 25-hydroxyvitamin D levels were also lower in HIV-subjects (16.4 range 0.7-74 ng/mL) than in controls (20 range 9–40.2 ng/mL) (*p* < 0.02).

Calcium and parathyroid hormone levels were not significantly different in the two groups of patients (data not shown).

### Correlation between lumbar spine and femoral neck DXA T-scores with demographic and environmental risk factors and calcium intake in HIV-infected subjects

The correlations between the studied risk factors and lumbar spine and femoral neck DXA T-scores are presented in Table 2. Age was negatively correlated with BMD measured in both the lumbar spine and femoral neck (*p* < 0.05). Higher BMI values were positively correlated with BMD values measured in the femoral neck (*p* < 0.05).

**Table 2:** Correlation between lumbar spine, femoral neck DXA T-scores and the studied parameters in the HIV-infected patients

	**Lumbar spine DXA T-score** **n = 112**	**P <**	**Femoral neck DXA T-score** **n = 112**	**P <**
**DEMOGRAPHIC AND LIFESTYLE CHARACTERISTICS**				
**Age**^**a**^	-0.20	**0.05**	-0.19	**0.05**
Female sex ^b^	0.05	ns	0.07	ns
BMI (kg/m^2^) ^a^	0.07	ns	0.11	ns
**BMI score**^**b,1**^	0.14	ns	0.20	**0.05**
Smoking status ^b,2^	-0.01	ns	0.09	ns
Free time physical activity ^b^	0.14	ns	0.06	ns
Heavy workers^b^	0.02	ns	0.04	ns
Italian espresso caffeine intake ^b,3^	-0.04	ns	-0.05	ns
Alcohol^b^	-0.03	ns	0.02	ns
Diabetes^b^	-0.14	ns	-0.02	ns
Postmenopause^b^	-0.17	ns	-0.17	ns
Fracture^b^	-0.05	ns	0.17	ns
**CALCIUM INTAKE**				
Daily calcium intake (mg/day)^a^	0.09	ns	0.05	ns
Dairy intake (times/week) ^a^	0.09	ns	0.05	ns
Daily milk intake (ml/day)^a^	0.07	ns	0.08	ns
Parmesan intake^b^	0.08	ns	0.15	ns
**Yogurt intake (ml/week)**^**b**^	0.23	**0.04**	0.11	ns
Dairy quartile intake (times /week)^b,4^	0.05	ns	0.04	ns
**BIOCHEMICAL PARAMETERS**				
Calcium (mg/dL)^a^	0.12	ns	0.08	ns
Phosphorus (mg/dL)^a^	0.09	ns	0.13	ns
Parathyroid hormone (pg/mL)^a^	-0.16	ns	-0.13	ns
25-Hydroxyvitamin D (ng/mL)^a^	-0.05	ns	-0.08	ns
**Bone alkaline phosphatase (IU/L)**^**a**^	-0.34	**0.002**	-0.32	**0.005**
**CHARACTERISTICS OF HIV INFECTION**				
**Drug addiction**^**b**^	-0.19	**0.05**	-0.25	**0.02**
CD4+ T-cell count (cells/μL)^a^	0.09	ns	0.12	ns
**Nadir CD4+ <200 (cells/μL)**^**b**^	-0.22	**0.05**	-0.23	**0.04**
Undetectable HIV-RNA^b^	0.07	ns	0.09	ns
**Time since HIV diagnosis**^**a**^	-0.17	ns	-0.25	**0.01**
**Score of ART exposure**^**b,5**^	-0.23	**0.002**	-0.34	**0.0001**
**Score of PI exposure**^**b,5**^	-0.20	**0.05**	-0.32	**0.001**
Duration tenofovir exposure > 1 year^b^	-0.12	ns	-0.11	ns
NNRTI exposure^b^	-0.01	ns	-0.01	ns
**HCV infection**^b^	-0.30	**0.001**	-0.27	**0.005**
Duration tenofovir exposure > 1 year^b^	-0.12	ns	-0.11	ns
NNRTI exposure^b^	-0.01	ns	-0.01	ns
**HCV infection**^b^	-0.30	**0.001**	-0.27	**0.005**

a: Pearson’s correlation test; b: Spearman’s rank correlation test.

^1^ BMI score = 1: **<** 25; 2: ≥ 25–29; 3: ≥ 30 (kg/m^2^).

^2^ Smoking habits were graded 1: non- smokers, 2 smokers, 3 former smokers.

^3^ Italian espresso caffeine intake = > 4 cups per day.

^4^ Dairy quartile intake (times /week) = 1: ≤8, 2: 9–10, 3: 11–14, and 4: ≥15.

^5^ ART and PI score = 0: no exposure; 1: < 5; 2: 5–9; 3: ≥ 10 years.

No correlation was found between daily calcium intake (specifically milk), Parmesan, weekly consumption or quartile calcium intake and BMD measured in either the lumbar spine or femoral neck. Yogurt intake was positively correlated with BMD measured in the lumbar spine (*p* < 0.04).

By stratifying patients into two groups (milk or milk + yogurt) we found that osteopenia and osteoporosis were significantly higher in the patients with milk only than in those with milk + yogurt (χ_MH=_^2^ 5.6; *p <* 0.02).

As regards biochemical markers, increasing values of bone alkaline phosphatase were negatively correlated with lumbar spine and femoral neck DXA T-scores (*p* < 0.002; *p* < 0.005, respectively).

### Correlation between lumbar spine and femoral neck DXA T-scores and characteristics of HIV- infection

Duration of HIV-infection was negatively correlated with BMD in the femoral neck (*p* < 0.01). BMD in both lumbar spine and femoral neck DXA T-scores was negatively correlated with drug addiction (*p* < 0.04; *p* < 0.02, respectively), nadir CD4+ T-cell count < 200 (cells/μL) (*p* < 0.05; *p* < 0.04, respectively), ART exposure score (*p* < 0.002; *p* < 0.0001, respectively), PI exposure score (*p* < 0.05; *p* < 0.001, respectively) and HCV co-infection (*p* < 0.001; *p* < 0.005, respectively) (Table 2).

The mean of the 10-year risk of fracture, calculated in 76 HIV-infected subjects, was 1.2% for hip fracture and 4.7% for major osteoporotic fracture.

**Figure 1:**
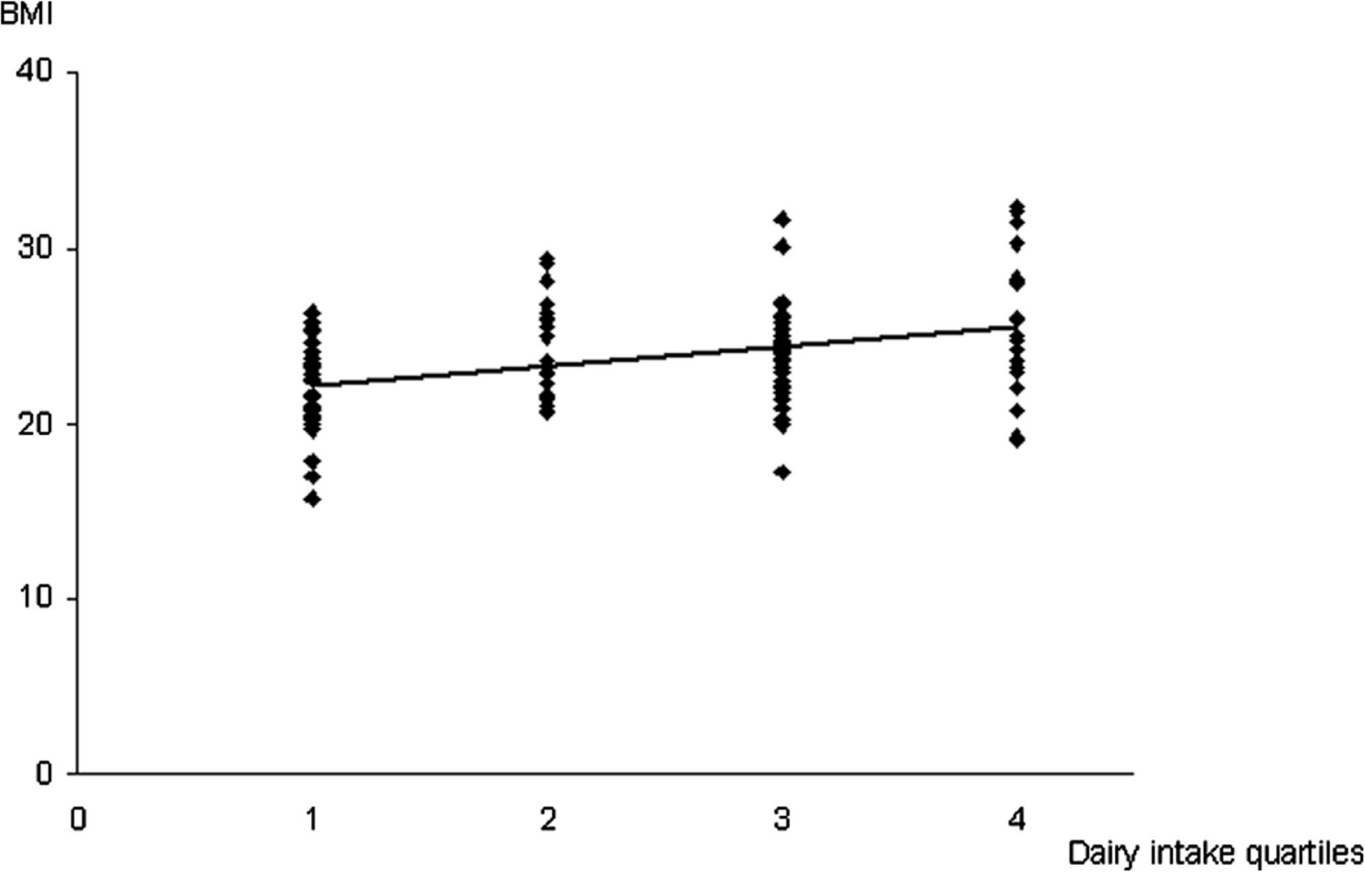
**Correlation between BMI and dairy intake quartiles (times/week).** The figure shows the significant Spearman’s correlation between BMI values and dairy intake quartiles (1: ≤8, 2: 9–10, 3: 11–14, and 4: ≥15) in the HIV-infected subjects (ρ = 0.36; *P* < 0.0001).

Figure 1 shows the positive correlation between BMI values and quartiles of dairy intake (ρ = 0.36; *p* < 0.0001). Lower dairy intake quartiles were more frequent in subjects with osteoporosis than without, although the difference was not significant (ρ = −0.17; *p* = ns).

No correlations between calcium intake and overall fracture risk at 10 years (r = −0.12; *p* = ns) and between calcium intake and risk of hip fracture at 10 years were found (r = −0.13; *p* = ns).

At multiple linear regression analysis, age and HIV/HCV co-infection were negatively correlated with BMD measured in the lumbar spine (*p* < 0.0001; *p* < 0.005, respectively), while an independent positive correlation was found between yogurt intake and BMD in the same site (*p* < 0.05). In the femoral neck T-score, age, nadir CD4+ T-cell count < 200 (cells/μL) and drug addiction (*p* < 0.01; *p* < 0.05; *p* < 0.0001, respectively) were negatively correlated with BMD (Table 3).

**Table 3:** Multiple linear regression analysis of factors correlated with lumbar spine and femoral neck DXA T-scores

**Variables**	**Lumbar spine DXA T-score**^**a**^		**Femoral neck DXA T-score**^**b**^	
	**β-coefficient**	**P <**	**β-coefficient**	**P <**
**HIV/HCV co-infection**	−1.3	**0.0001**	**_**	
**Age**	−0.48	**0.005**	−0.48	**0.01**
**Yogurt intake**	0.3	**0.05**	**_**	
**nadir CD4+ T-cell count < 200 (cells/μL)**	**_**		−0.32	**0.05**
**Drug addiction**	**_**		−1.4	**0.0001**

^a^ Variables excluded from the model for lumbar spine DXA T-score assessment: bone alkaline phosphatase, drug addiction , time since HIV diagnosis, score of ART and PI exposure.

^b^ Variables excluded from the model for femoral neck DXA T-score assessment: BMI score, Bone alkaline phosphatase, time since HIV diagnosis, score of ART and PI exposure, HIV/HCV co-infection.

## Discussion

To our knowledge, this is the first investigation on calcium intake and its relationship with BMD in adult subjects with HIV-infection. Previous studies in this area focused on calcium intake and its potential metabolic impact in HIV-subjects, or on its link with BMD in the subset of a HIV- pediatric population [21-23]. Despite the typical southern Italian regional habit of drinking a glass of milk with coffee in the morning (about 300 mg of calcium), median daily calcium intake in our HIV-infected subjects was lower than the recommended daily amount, as reported by other Asian and European studies [24-26]. Milk is a good source of both calcium and vitamin D but other calcium-rich foods are needed to create a balanced diet assuring sufficient calcium and vitamin D intake in an HIV-population.

Parmesan cheese could be included in the "Mediterranean diet group" of foods because it is usually added to pasta and pizza. In fact, in our study interview Parmesan (25 g = 300 mg calcium) was regularly consumed in 90% of the HIV-subjects.

Among the various dairy foods available we considered yogurt, which contains live active cultures and a substantial amount of calcium and vitamin D. Interestingly, we found that weekly yogurt intake was a protective independent predictor of BMD in the lumbar spine.

When the patients were divided into two groups the analysis showed a more favorable relationship between yogurt plus milk and BMD than milk alone. No previous study has examined the relationship between yogurt intake and BMD in HIV-infected subjects and only Sahni et al. [27] showed that milk and high yogurt intake were positively associated with hip and spine BMD in immunocompetent subjects in the Framingham Offspring Study.

Probiotic microorganisms have considerable immunomodulatory effects in the gut-associated lymphoid tissue [28]. The importance of the gut microbiota for the development of the host’s immune system and the suggested connection between gut microbiota and bone mass in mice led the researchers to investigate the impact of probiotic yogurt on bone mass, especially in the HIV- population [29]. Further studies are required to evaluate the gut microbiota and other micronutrients as a novel therapeutic target for osteoporosis [30].

In the present study the prevalence of osteoporosis was significantly higher in HIV- infected than in uninfected subjects, a result similar to previous meta-analyses [31].

Currently, more intensive investigations have been made to better understand whether HIV infection *per se* is a risk factor for low BMD [1,7]. Since the virus itself may induce hypovitaminosis D through many mechanisms (increased levels of TNF-α, inhibition of renal hydroxylation and induction of 25-hydroxyvitamin D consumption by the macrophages and lymphocytes) [7,32,33], the abnormal biochemical features found in our HIV-infected subjects compared with the controls, in particular the lower vitamin D levels, may support the hypothesis that HIV *per se* might play a role in the pathogenesis of bone loss. In addition, this finding in a sunny country like Italy might suggest the need to develop and recommend new key steps of calcium and vitamin D intake in the prevention of osteoporosis.

Several HIV-related factors were correlated with BMD on univariate analysis in both the lumbar spine and femoral neck in our HIV-infected subjects. The correlation between time of HIV diagnosis and BMD in the femoral neck as well as nadir CD4+ T-cell count < 200 cells/μL and BMD in both the lumbar spine and femoral neck and the finding that nadir CD4+ T-cell count < 200 cells/μL was an independent predictor of low BMD, suggest that HIV infection *per se* and in particular the more advanced stages of the disease might affect the skeletal system. This finding also suggests that an earlier commencement of ART might have a beneficial effect on BMD.

On the other hand, we found a significant negative correlation between longer duration of ART, especially PI exposure, and both lumbar spine and femoral neck BMD on univariate analysis.

How to strike a balance between the various aspects to be considered when providing ART, (such as when treatment should be commenced or the classes of drugs to be used, its influence on the progression of HIV disease and, in the present case, its effects on BMD) in order to maximize its benefits is an ongoing debate. In this respect, while Grund et al. [34] showed that BMD continuously declined in a HIV-group receiving continuous ART, Bolland et al. [35] found no evidence of accelerated bone loss over 6 years in middle-aged HIV-infected men treated with ART [36]. Many studies on the influence of ART have shown that the relative risk of low BMD is greater when PI are used [31,37-39], in agreement with our results. Unfortunately, we were unable to evaluate which drugs of the PI class were responsible for the low BMD, whereas other authors have shown that indinavir inhibits bone formation and lopinavir/ritonavir and atazanavir/ritonavir are associated with BMD loss [31,39].

We did not find a significant correlation between tenofovir exposure > 1 year and BMD in HIV-subjects, unlike previous reports [39,40]. Recent data showed that the introduction of tenofovir or emtricitabine/tenofovir was associated with a 0.8 %–1.1 % decrease in BMD which occurred mainly within the first year in high-risk HIV-infected individuals [41]. In our study the small number of enrolled HIV subjects and the long exposure to different antiretroviral drugs made it difficult to show the real impact of tenofovir exposure on BMD health.

Moreover, no significant correlation was found between the small number of fractures reported in our HIV- subjects and BMD at either of the sites. In a recent Italian study, metabolic factors such as BMI and diabetes mellitus were independently correlated with vertebral fractures in HIV-infected patients [42]. Although low BMD in the setting of HIV-infection is frequently observed, data on its relationship with fracture rate are still under investigation [43].

To improve the ability to predict subsequent fragility fracture in our patients, we used the WHO FRAX equation [20]. Although fracture risk at ten years and calcium intake showed an inverse relationship this was not statistically significant. However, the role of the FRAX score in the HIV-population is still under debate because the HIV-independent risk factors, HIV-related parameters and ART characteristics were not evaluated by the score itself [44].

Similar to other studies on enrolled HIV-infected subjects with a long duration of infection, we found that older age and BMI values had a negative or positive correlation with BMD, respectively [45-47]. In this respect, a meta-analysis on ten studies showed that low BMI was the main factor behind the lower BMD values in HIV-infected than in normal uninfected individuals [47].

The results regarding the relationship between dairy product consumption and BMI are contradictory [48]. However, some cross-sectional data suggest that lower-fat dairy products such as milk and yogurt are associated with lower adiposity [48]. The positive correlation between higher BMI values and BMD in the femoral neck DXA T-score or the increasing quartiles of calcium intake, suggest a protective role for calcium intake in the prevention of BMD loss in our HIV-subjects [49]. This observation may also encourage physicians to recommend an increase in calcium consumption especially in HIV-subjects with low BMD.

However, the ideal daily calcium intake to prevent osteoporosis in HIV-positive subjects is not clear because it depends not only on known general and environmental risk factors but also on the patient’s long-term immuno-virological course.

For most people, a daily calcium intake of between 1000 and 1300 mg is both safe and potent but we hypothesize that the dose should be reviewed in relation to ART and HIV-status.

Recent data have suggested that patients with AIDS and addicted to heroin might be at particular risk for bone loss [50]. The independent negative correlation found between drug addiction and low BMD in our HIV subjects confirms this finding.

Another interesting result emerging from our study is the independent correlation between HIV/HCV co-infection and BMD.

Vitamin D deficiency was found in HIV/HCV co-infected patients [51]. Moreover, higher vitamin D levels were independently associated with a rapid virological response in patients with genotype 1 chronic hepatitis C [52]. Increasing vitamin D intake may positively modulate the response to antiviral treatment in HCV-infected or HIV/HCV co-infected patients. Further research is necessary to better understand the impact of viral hepatitis on BMD in HIV-infected and uninfected subjects and discuss the clinical applications and future direction of this field [53].

## Conclusions

To our knowledge, this is the only study to date to evaluate calcium intake and low BMD in HIV-infected subjects. Two interesting findings emerge from our study, firstly, among the foods rich in calcium yogurt was an independent protective predictor of BMD in HIV-subjects; second, HIV/HCV co-infection, drug addiction and nadir CD4+ T-cell count < 200 cells/μL were independent predictors of severe bone disease. It seems essential to implement medical treatment by promoting behavioral changes in food intake and lifestyle in the primary prevention of bone disease in chronically-infected people.

## Abbreviations

HIV, human immunodeficiency virus; BMD, bone mineral density; TNF-α, tumor necrosis factor-α ART, antiretroviral therapy; PI, protease inhibitors; NNRTI, non-nucleoside reverse-transcriptase inhibitors; BMI, body mass index; DXA, dual-energy radiographic absorptiometry; AIDS, acquired immunodeficiency syndrome; Vs, versus.

## Competing interests

The authors declare that they have no competing interests with respect to this article.

## Authors’ contributions

VLV and PDC designed the study and drafted the manuscript. MS participated in the design of the study and performed the statistical analysis. LG, GM, SLS, CC and SM set up and conducted the study in the field and contributed to the interpretation of the results. GC carried out the DXA. FT carried out the laboratory analysis. The ethics applications and funding applications were submitted by PM and PDC as principal investigators in the control group. GM and PDC conceived the study, and participated in its design and coordination. All authors read and approved the final manuscript.

## Pre-publication history

The pre-publication history for this paper can be accessed here:

http://www.biomedcentral.com/1471-2334/12/192/prepub
